# Diagnosis of a brain stroke using wideband microwave scattering

**DOI:** 10.1098/rsos.221560

**Published:** 2023-03-22

**Authors:** Dastan Ismail, Samah Mustafa

**Affiliations:** Electrical Engineering, Salahaddin University-Erbil, Kurdistan region, Iraq

**Keywords:** microwave monitoring system, wavelet transform, wavelet matched filters, wavelet energy, wavelet Shannon entropy

## Abstract

This paper presents a new computer-aided microwave monitoring system as a promising, portable and inexpensive tool to detect and localize brain stroke using a bank of a new wavelet-matched filters. The head is exposed to microwave radiation over the band from 1.1 to 3 GHz, and the backscattered signals at a hemi-elliptical array of 16 antenna elements surrounding the head are filtered to analyse the perturbation in the microwave signals from the brain. A novel technique is applied to remove the strong reflection from the air–skull interface as a way to estimate the target response and is compared with different techniques from literature to portray their role in the performance. The study results approve that the intensity and the distribution of wavelet energy and Shannon wavelet entropy in the filtered microwave signal, and the novel tool based on the distance between the wavelet energies at symmetric opposite antennas are promising candidate signatures for computer-aided detection and localization of a stroke.

## 1. Introduction

Brain stroke is a major health-related challenge and it affects approximately 16 million individuals worldwide annually [1]. Brain stroke usually leads to cognitive impairments that compromise functionality and may cause death in many cases. Early diagnosis and prompt treatment are crucial to reduce brain damage and other complications. Stroke diagnosis relies on a medical history and physical and neurological examination, and primarily relies on brain imaging using a computed tomography (CT) scan or magnetic resonance imaging (MRI) [1]. These tests produce high-resolution imaging. However, the tests are expensive and the equipment is non-portable and not always accessible, especially in rural medical clinics.

Researchers have the potential to detect brain stroke and breast cancer using microwave technologies based on the difference in the electrical properties of healthy and defective tissue references in the breast and brain. Microwave is proven a safe, cost-effective and reliable signal detection method. However, stroke detection entails numerous challenges primarily because of the complex and rich structure of the human brain, the nature of stroke as a disturbance in the blood supply and the urgent need for diagnosis and intervention [2,3]. Therefore, the goal is to develop a portable and inexpensive microwave system that can be carried by first-responder paramedics.

Microwave imaging has been applied for brain stroke detection [4–7]. The method is computation intensive and time consuming, and it faces significant challenges to achieve satisfactory and reliable results; furthermore, computationally demanding algorithms are used to process large numbers of collected signals comprehensively in a short time [2,8,9]. Therefore, a microwave monitoring system, based on pattern recognition of the scattered signal, has been investigated for detection purposes.

The authors in [10] set up a microwave prototype system to collect the reflected signals and applied a subspace classifier method to detect haemorrhagic stroke and differentiate it from ischaemic stroke. Another subspace classifier was applied by [11] on backscattered signals observed using the numerical analysis finite-difference time-domain (FDTD) and two-dimensional computational domain. However, these classification methods rely on scattered data of high-dimensional spatial and frequency microwave measurements. In [8], backscattered signals from a healthy and defected object were determined using two-dimensional FDTD, such that stroke detection depends on the similarity of two reflected signals at the same antenna positions with a threshold. In [12], strong reflection from the air–head interface was ideally removed using a backscattered signal from a healthy brain and afterwards, signal classification was performed for stroke detection. Large-dimensional spatial microwave signals are classified according to their signal amplitude distribution. However, the instantaneous amplitude and the classification are expected to be sensitive to an error in background reflection removal. Three-dimensional FDTD simulation was performed in [9] to collect the backscattered signal over 3 GHz bandwidth, and the background reflection was removed using a reference signal collected upward of the position of interest. The scattered signals were processed using a wavelet-matched filter based on a Gaussian-modulated sinusoidal signal to detect a stroke. A review of machine learning techniques and features used for stroke diagnosis and classification is presented in [1]. However, these studies rely on MRI and CT scans. In [13], two deep neural networks were designed and configured to detect and localize brain stroke in two-dimensional head model. The authors used S-parameter measurements of wideband antenna array to learn about the dielectric signature of stroke from a large simulated cohort.

In this study, a bank of new wavelet-matched filters is built and used to process the scattered signals and detect brain stroke. A novel approach to remove the strong backscattered signal from the air–skin interface is introduced. The proposed approach is compared with other approaches to highlight its efficiency. The scattered signals were experimentally collected using the microwave system in [14]. The system operates across a wide band of microwave frequencies of 1.1–3 GHz.

The paper is organized into six sections. The second section briefly describes the microwave system that is used to collect the scattered signals, followed by the processing to estimate the target response in the next section. The wavelet-matched filters are presented in the fourth section, and the results using different metrics are presented in the fifth section. The study is concluded in the last section.

## 2. Microwave measurement system

**Figure 1.**
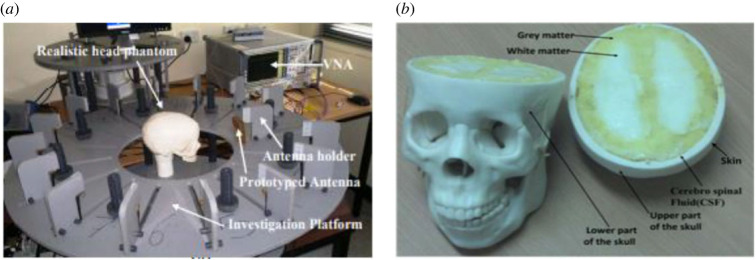
(*a*) Microwave system for brain stroke detection, (*b*) head phantom [15].

As shown in figure 1, a three-dimensional head phantom was used as a part of the measurement system. It consisted of the three main brain tissues: cerebral fluid, white matter and grey matter, in addition to the skull. The tissues were fabricated as a combination of few materials as described in [4], to present the electrical properties described clearly in [15]. An ellipsoidal object with electrical properties of blood and dimensions of 2 × 2 × 1 cm^3^ was inserted in the head at different positions to represent a brain stroke. An array of 16 antennas were uniformly distributed around the head and connected to the Rohde and Schwarz ZVA24 vector network analyser (VNA) to allow sequential measurement of each antenna's backscattered signal using a mono-static radar approach. Each antenna in the array radiates a discrete set of 401 equidistant frequencies over the band 1.1–3 GHz and then captures the reflected signal. The design and configuration parameters of the used antenna are presented in [14], and the details of the remaining microwave measurement set-up are available in [4]. The measurements data were collected at the school of ITEE in the University of Queensland, Brisbane, where the microwave system was designed and built.

## 3. Signal pre-processing to remove the background reflection

The early reflection of the head–air interface and the later background clutter cause a strong background reflection that dominates the backscattered signal. Furthermore, the background reflections are different in the spatial domain because of the hemi-ellipsoidal shape of the head and its heterogeneity [16]. Pre-processing of the backscattered signal to eliminate the dominant background reflection and extract the brain tissues' response is therefore crucial for detecting and localizing a target in the brain. Different approaches have been applied previously to find a target response, as described here:

### 3.1. First approach App-1

**Figure 2.**
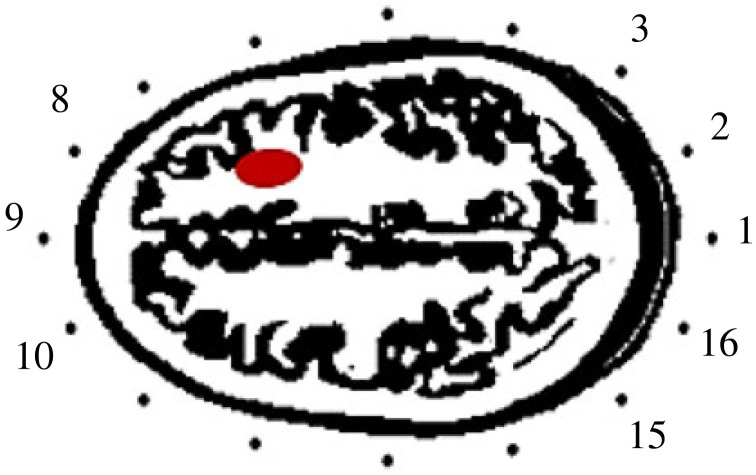
16 antenna elements arranged in a two-dimensional array around the head with a stroke represented by red.

Under the assumption of existing medical record, specifically the backscattered signals of a healthy brain of a patient, an ideal target response at an antenna location is extracted by subtracting the recorded signal of the healthy brain from the signal of the defected brain for the same patient and antenna location [9,17]. However, it is not a realistic scenario. In figure 2, L = 16 antenna array elements are distributed around the head, and the response of interest at a point is observed as

3.1 $$\begin{array}{rl} & \delta_{i}=\delta_{i}^{\mathrm{target}}-\delta_{i}^{\mathrm{notarget}}\\ & i=1,2,3,\ldots ,L \end{array}$$

### 3.2. Second approach App-2

Rotation removal is performed where the target response is extracted by subtracting the signals of slightly displaced adjacent antenna locations belonging to the same set of backscattered signals [16,17] as given by

3.2 $$\delta_{i}=\{\begin{array}{ccc} \delta_{i}^{\mathrm{target}}-\;\delta_{i-1}^{\mathrm{target}} & \mathrm{if} & i=2,3,\ldots ,L\\ \delta_{1}^{\mathrm{target}}-\;\delta_{L}^{\mathrm{target}} & \mathrm{if} & i=1 \end{array}$$

### 3.3. Third approach App-3

Because of a natural head symmetry with respect to a central line dividing the brain into right and left halves, the background reflection can be removed by subtracting the signals of two antennas placed symmetrically opposite [16–18]. Based on the antennas’ order around the head as shown in figure 2, the target response is observed as

3.3 $$\delta_{i}=\delta_{i}^{\mathrm{target}}-\delta_{\left(L+2-i)\mathrm{mod}(L+1\right)}^{\mathrm{target}}$$

Using an array of *L* antennas distributed uniformly around the head, *L*/2 target responses are determined since

3.4 $$\delta_{i}=-\delta_{\left(L+2-i)\mathrm{mod}(L+1\right)}$$

It is not possible to localize the target, whether it is in the right or left half, unless further processes are applied. In [16], the negative values in the target response are clipped to zeros to distinguish the opposite extracted responses and to collect *L* targets signals around the head.

### 3.4. Fourth approach App-4

It is based on the average removal where the background reflection is determined either as the average of the backscattered signals from a number of antennas located close to the position of interest [17], or as the average of all backscattered signals [19].

3.5 $$\delta_{i}=\delta_{i}^{\mathrm{target}}-\mathrm{avg}[\delta_{j}^{\mathrm{target}}],$$

where *j* denotes the index of the antennas that are considered in the average estimation. However, the hemi-ellipsoidal shape of the head and its heterogeneity may degrade the effectiveness of this approach.

### 3.5. Fifth approach App-5

This work introduces a novel approach to estimate the response of interest. The background reflection at a specific antenna position is observed either as the average of the adjacent and the opposite two scattered signals or as the average of only two opposite scattered signals. The target response is constructed as given by

3.6 $$\delta_{i}=\delta_{i}^{\mathrm{target}}-\mathrm{avg}[\delta_{i+1}^{\mathrm{target}},\delta_{\left(L+2-i)\mathrm{mod}(L+1\right)}^{\mathrm{target}},\;\delta_{\left(L+1-i)\mathrm{mod}(L+1\right)}^{\mathrm{target}}]$$

or

3.7 $$\delta_{i}=\delta_{i}^{\mathrm{targe}t}-\mathrm{avg}[\delta_{\left(L+2-i)\mathrm{mod}(L+1\right)}^{\mathrm{target}},\delta_{\left(L+1-i)\mathrm{mod}(L+1\right)}^{\mathrm{target}}]$$

such that $\;\delta_{i}\neq -\delta_{\left(L+2-i)\mathrm{mod}(L+1\right)}.$

To detect and localize the stroke and to optimize the performance of the processing in this work, the approaches were applied to remove the background reflection and determine the target scattered signal.

## 4. Signal post-processing and wavelet-matched filters

The measurements were made in the frequency domain where the antennas radiate an unmodulated carrier signal of equidistant frequencies sequentially over the 1.1–3 GHz spectrum. The backscattered signals were collected and transformed into a time domain using inverse Fourier transforms to be processed for diagnoses. After removing the background reflection, the target response was processed through a bank of wavelet-matched filters to analyse its contents in both the frequency (scale) and time domains and maximize the output of the signal in a noisy environment.

The set of radiated signals from each antenna had a rectangular pulse shape in the frequency domain over the defined spectrum and a sinc pulse in time domain that has a zero frequency response at zero frequency. Therefore, a new mother wavelet with a sinc shape was constructed to match with the radiated signal. As it is known, the sinc function plays a fundamental role in wavelet theory where the basic functions of the Shannon wavelets are defined by the sinc function.

The sinc function continues to both negative and positive infinities without dropping to zero amplitude. Therefore, it is truncated to a limited length symmetrically around the main lobe and the samples located out of this range are neglected. Next, the truncated pulse is shifted to the right to be represented by positive indexes similar to the scattered signal, as given by

4.1 $$\;\;\Omega (t)=\mathrm{rect}\left(\frac{t-(T/2)}{T}\right).\;\mathrm{sinc}(t)$$

and

4.2 $$\psi (t)=\Omega \left(t-\frac{T}{2}\right)$$

**Figure 3.**
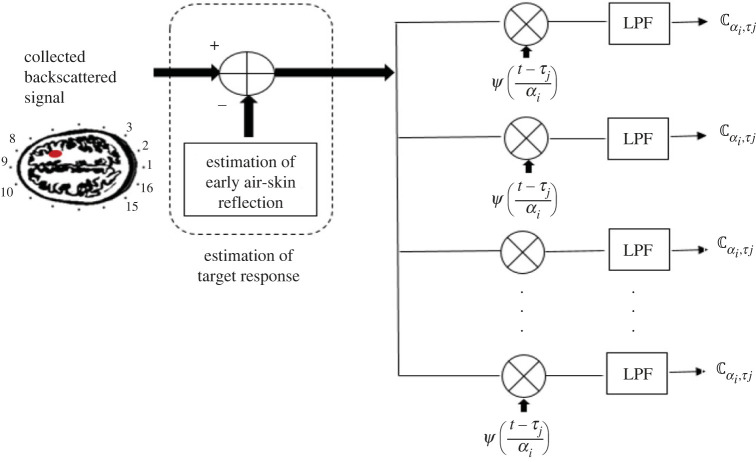
Bank of wavelet-matched filters to process the target response at each antenna position. LPF, low-pass filter.

To analyse the estimated target response and observe the oscillation of interest and where it happens, it is correlated with a set of scaled-time shifted wavelets (figure 3) and ψ(t) is used as mother wavelet with T samples as

4.3 $$\begin{array}{rl} C_{\alpha ,\tau }= & \int_{0}^{t}\delta (t)\;.\;\;\psi_{\alpha ,\tau }(t)\;dt\\ = & \int_{0}^{t}\delta (t)\;.\;\;\frac{1}{\sqrt{\alpha }}\psi \left(\frac{t-\tau }{\alpha }\right)\;dt. \end{array}$$

α denotes the scale or the inverse of the frequency and defines the way in which the wavelet filter response is stretched or squished to catch low- or high-frequency contents respectively. τ defines where the filter response is positioned in time; a smaller/larger offset will shift the position to the left/right. $C_{\alpha ,\tau }$ is the wavelet coefficient that represents the output of each matched filter. The filter bank serves as a wavelet transform to extract local spectral and temporal responses simultaneously. In this paper, the output of the filter bank around the head is evaluated using different metrics as scalogram and wavelet entropy at diverse reception points in the array.

Scalogram is a method to display the distribution of relative wavelet energy (RWE) in the scale–time plane, which portrays the probability distribution of energy as a function of time and scale (frequency) [10]. It is given by

4.4 $$E_{\tau ,\alpha }=\frac{|C_{\alpha ,\tau }{|}^{2}}{\sum_{\tau }\sum_{\alpha }|C_{\alpha ,\tau }{|}^{2}}$$

Shannon wavelet entropy is used to observe and diagnose the unsteadiness and complexity of the scattered signal in the time and scale domain that is built on wavelet analysis [10,20]

4.5 $$\epsilon_{\alpha }=\underset{\tau }{\sum }|C_{\alpha ,\tau }{|}^{2}\log_{2}|C_{\alpha ,\tau }{|}^{2}$$

4.6 $$\epsilon_{\tau }=\underset{\alpha }{\sum }|C_{\alpha ,\tau }{|}^{2}\log_{2}|C_{\alpha ,\tau }{|}^{2}$$

where $\epsilon_{\alpha }$ and $\epsilon_{\tau }$ denote the wavelet entropy in the scale and time domain, respectively.

## 5. Results

As described earlier, the 16 distributed antennas around the head phantom (figure 2) sequentially radiate a sinc signal over 1.1–3 GHz and collect the backscattered signal. In this section, the aforementioned approaches are applied independently to remove the strong reflection of the air–skull interface to evaluate and depict the impact of the pre-processing on the whole performance. The extracted responses are passed through the bank of wavelet-matched filters which are built in this work to analyse and diagnose a stroke emulated in the head phantom as depicted in figure 2. The filters' outputs are used to determine the distribution of wavelet energy in the scale–time plane at different spatial points around the head. We noticed a sparse energy in the wavelet domain and a difference in the number, intensity and time offset of the hot spots in the plane. The diagnosis of stroke depends on the variance in the results. The signals at antennas 2, 6, 7 and 11 are selected for the presentation here because of the limited space and the similarity of the results at the antennas that are located far from the target of interest.

### 5.1. Results based on App-1

**Figure 4.**
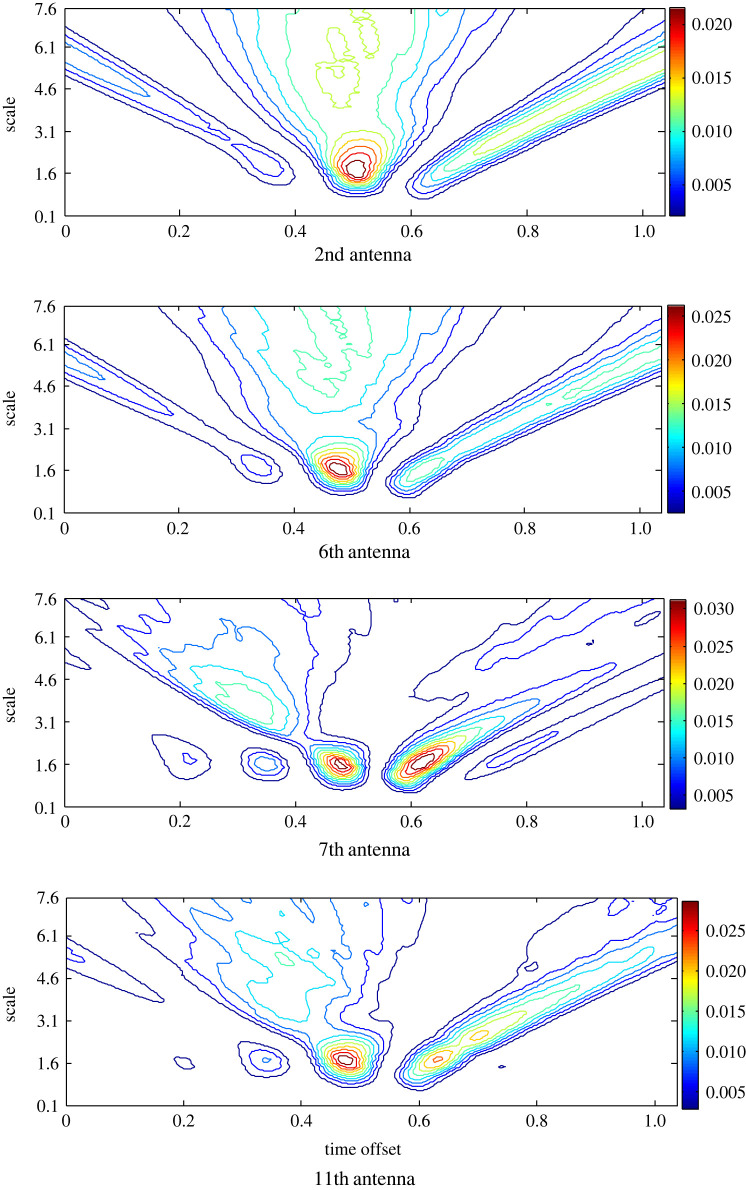
The scalogram of the matching filtered signals at antennas 2nd, 6th, 7th and 11th after the pre-processing based on APP-1 to estimate the target response.

The background reflection in the backscattered signals is removed using the ideal App-1 to estimate the target response around the head. The scalogram records the highest RWE at the 7th antenna with more than one hot spot (figure 4). Although the 6th antenna is close to the target, nearly equal RWE is recorded at the 11th antenna, which is located opposite the 7th antenna. Less RWE at an earlier time offset is recorded at the 2nd antenna that is far away from the emulated target. Based on the scalograms and the variance they portray at different antenna positions, the target is localized closer to the 7th antenna.

**Figure 5.**
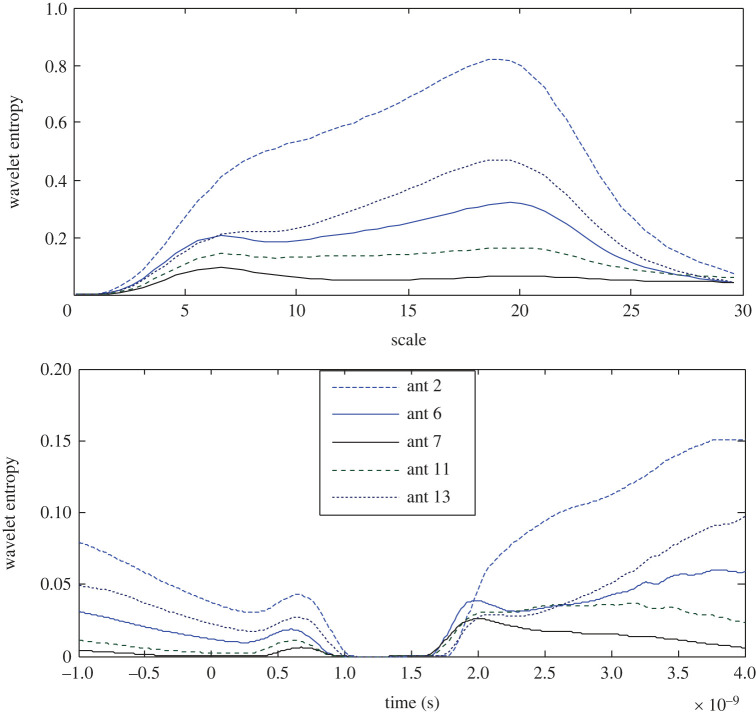
Wavelet Shannon entropy of the target response in the scale and time domain at 2nd, 6th, 7th, 11th and 13th antennas using APP-1.

Using equations (4.5) and (4.6), the wavelet entropy is observed as a function of the scale and time offset in the filter's response and is presented in figure 5. The highest RWE at the 7th antenna records the lowest entropy in both the scale and time domain. The next lower entropy is determined at the 11th antenna, i.e. facing the target in the opposite half of the brain, while the signal at the 2nd antenna, which is located far from the stroke, records higher entropy as an indication of an inverse relationship between the wavelet energy and wavelet entropy. Furthermore, a time slot of zero entropy can be observed in the time domain at all the antennas except for the 7th antenna where a couple of zero entropy slots are recorded. As illustrated in figure 5, the variance in the results states that the stroke is closer to the 7th antenna.

### 5.2. Results based on App-2

**Figure 6.**
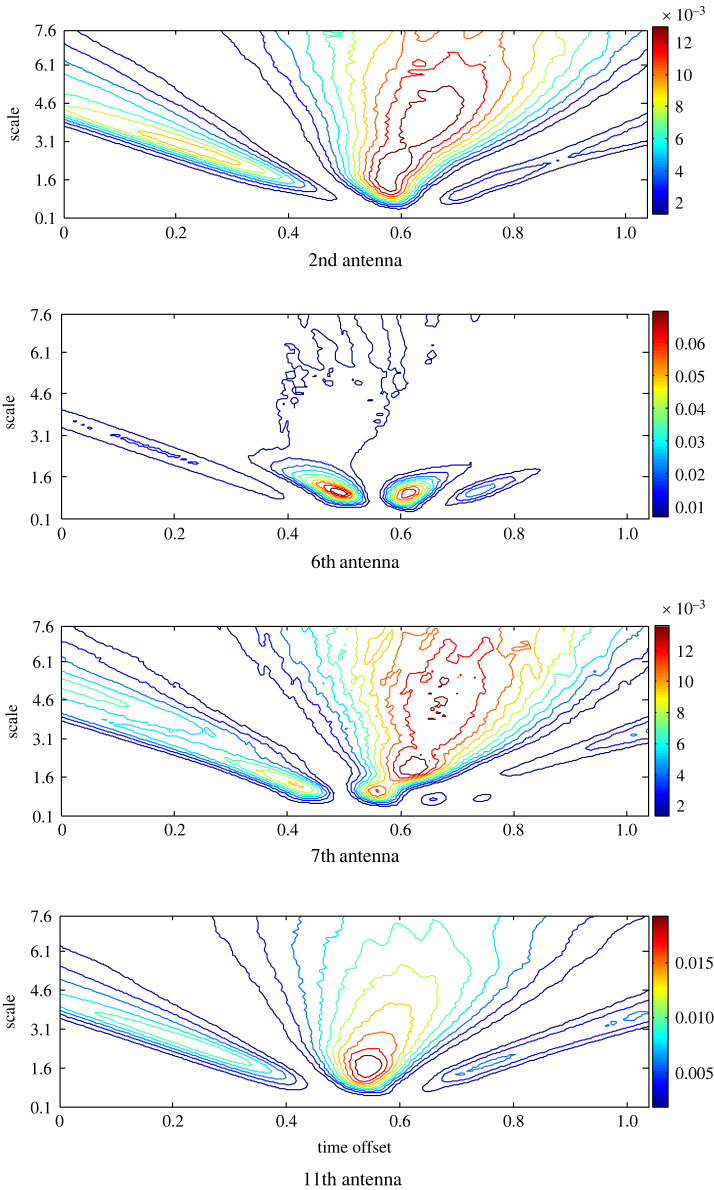
The scalogram of the matching filtered signals at the 2nd, 6th, 7th and 11th antennas after the pre-processing based on APP-2 to estimate the target response.

A similar analysis is applied to the estimated target response using App-2 (figure 6). The signals from the same antennas are presented for the diagnosis and comparison purposes. The signal of the 6th antenna has the highest RWE and two hot spots at two different time offsets over a limited range of the scales, while in the other scalograms, RWE is extended to larger scales. By contrast, unexpectedly, the 2nd antenna that is located far and the 7th one that is located close record the lowest RWE.

**Figure 7.**
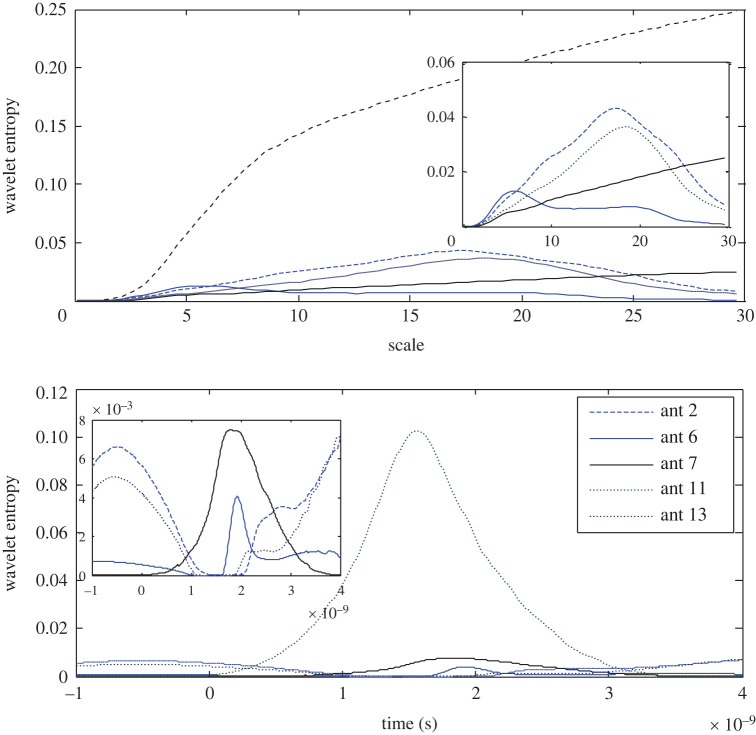
Wavelet Shannon entropy of the target response in the scale and time domain at the 2nd, 6th, 7th, 11th and 13th antennas using APP-2.

The results in figure 7 show that as a result of the noticed inverse relationship between the wavelet energy and entropy, the lowest wavelet entropy is observed at the 6th antenna; however, the lowest RWE does not necessarily result in the highest entropy, since the 11th antenna records the highest entropy in contrast to its state using App-1. A clear difference in the time slot of zero entropy around the head is observed. All the results except the dominator entropy of the 11th antenna are replotted to portray a clear sight on the other antennas. The variances in the scalograms and the wavelet entropy around the head indicate that the stroke exists closer to the 6th antenna.

Furthermore, the differences in the scalogram and wavelet entropy using App-2 compared with that determined using App-1 illustrate the impact of the background removal approach to estimate the stroke response and in turn the diagnosis process.

### 5.3. Results based on App-3

**Figure 8.**
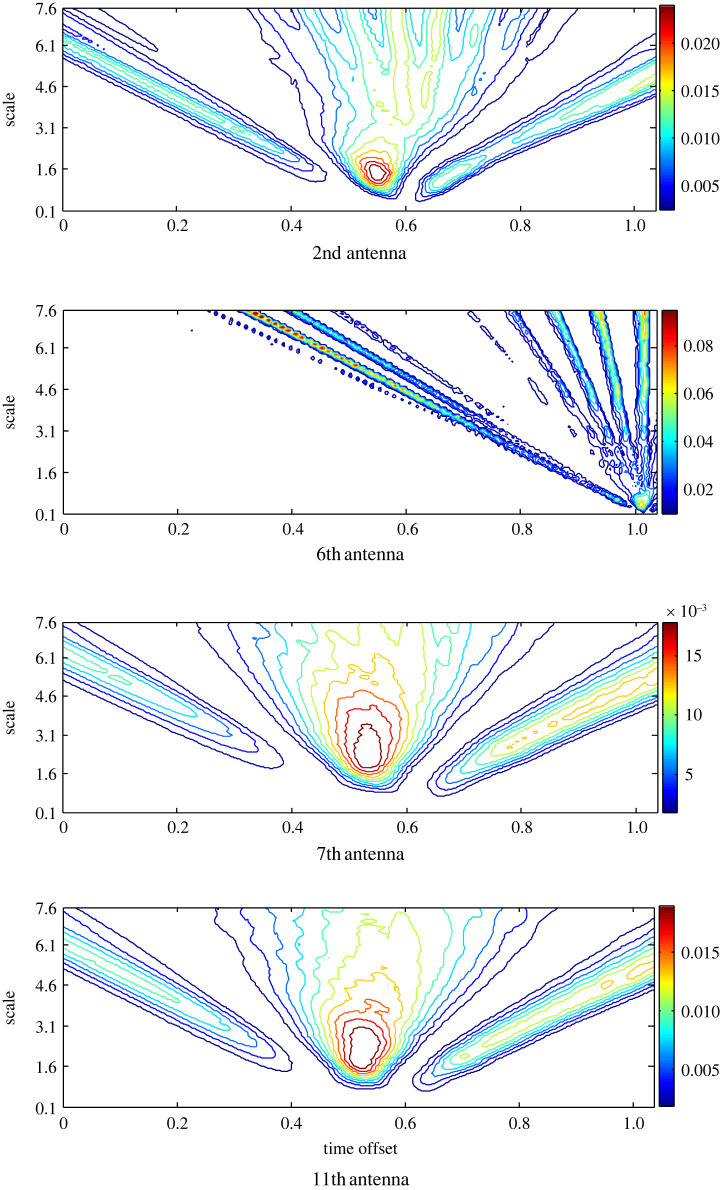
The scalogram of the matching filtered signals at the 2nd, 6th, 7th and 11th antennas after the pre-processing based on APP-3 to estimate the target response.

The target response is determined using App-3 and the scalogram of the matched filters’ output is presented in figure 8. A completely different distribution is obtained, where the highest RWE is found at the 6th antenna, and the lowest RWE is recorded by the oppositely located antennas 7 and 11.

**Figure 9.**
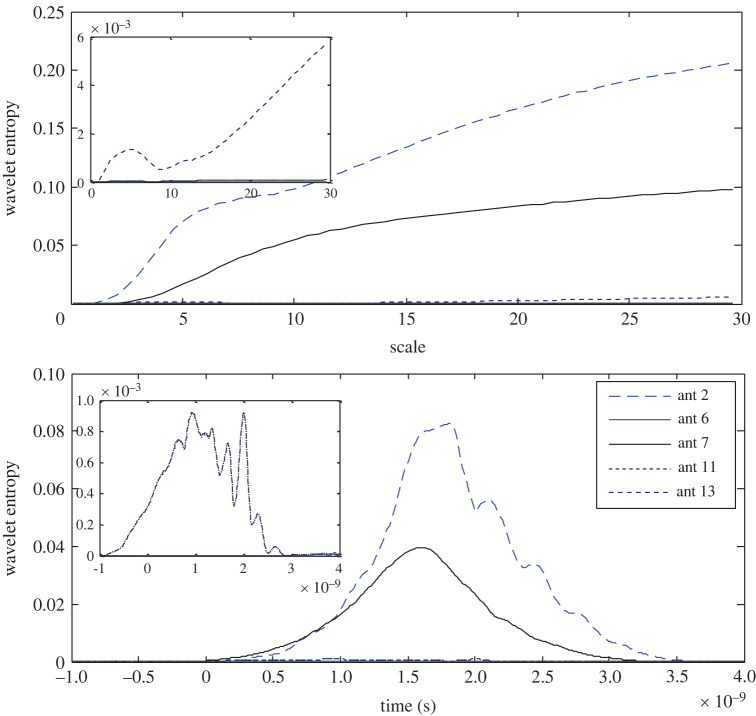
Wavelet Shannon entropy of the target response in the scale and time domain at the 2nd, 6th, 7th, 11th and 13th antennas using APP-3.

The wavelet Shannon entropy is presented in figure 9. The three and two lowest entropies are zoomed in in the scale and time domains, respectively, to show the extremely low entropy as it is recorded at the 11th antenna and nearly zero entropy at the 6th antenna in both domains. Similarly, the highest RWE at the 6th antenna is accompanied by the lowest entropy and the lowest RWE does not necessarily result in the highest entropy. Unexpectedly, the 2nd antenna records the highest Shannon entropy in the scale and time domains. The diagnosis process using App-3 refers to the 6th antenna as the closer to the stroke.

### 5.4. Results based on App-4

**Figure 10.**
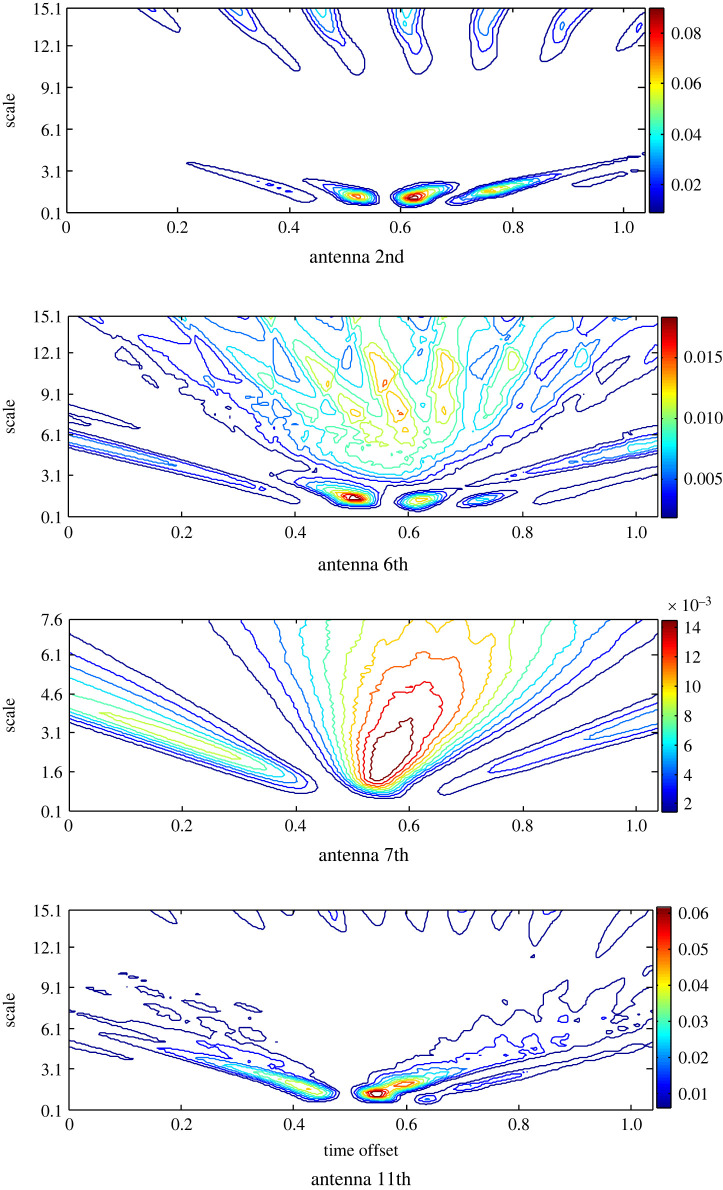
The scalogram of the matching filtered signals at the 2nd, 6th, 7th and 11th antennas after the pre-processing based on APP-4 to estimate the target response.

The target response based on App-4 is similarly processed and transformed into the wavelet domain. Although the 2nd antenna is located far from the stroke, it records the highest RWE and observes more than one hot spot, as shown in figure 10. In addition, the 11th antenna in a symmetric opposite position to the 7th antenna records high RWE and more than one hot spot. The scalograms of the closer 6th and 7th antennas cover large scales in contrast to the other scalograms. Further, the scalogram of the 7th antenna causes a single hot spot with evidently the lowest RWE. The variance in the scalograms' shape around the head points to a stroke located close to the 7th antenna, although it records the lowest RWE.

**Figure 11.**
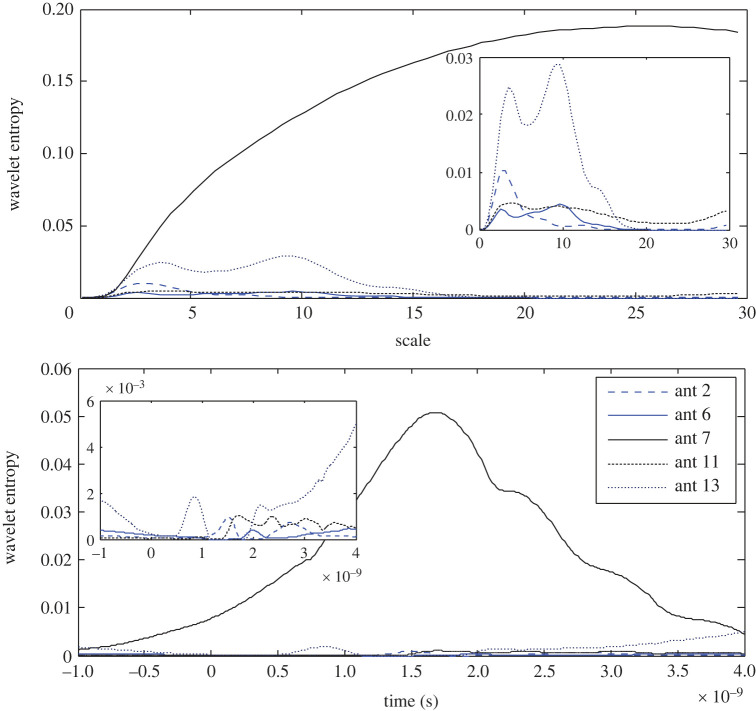
Wavelet Shannon entropy of the target response in the scale and time domain at the 2nd, 6th, 7th, 11th and 13th antennas using APP-4.

By contrast to the results of the earlier approaches, the wavelet entropy at the 7th antenna is the highest in both the scale and time domains as expected as a result of the lowest RWE. While the 6th antenna records the lowest entropy (figure 11). The wavelet entropy at the 2nd, 6th and 11th antennas are zoomed in to illustrate the difference in the results, where a slot of zero entropy is observed.

### 5.5. Results based on App-5

**Figure 12.**
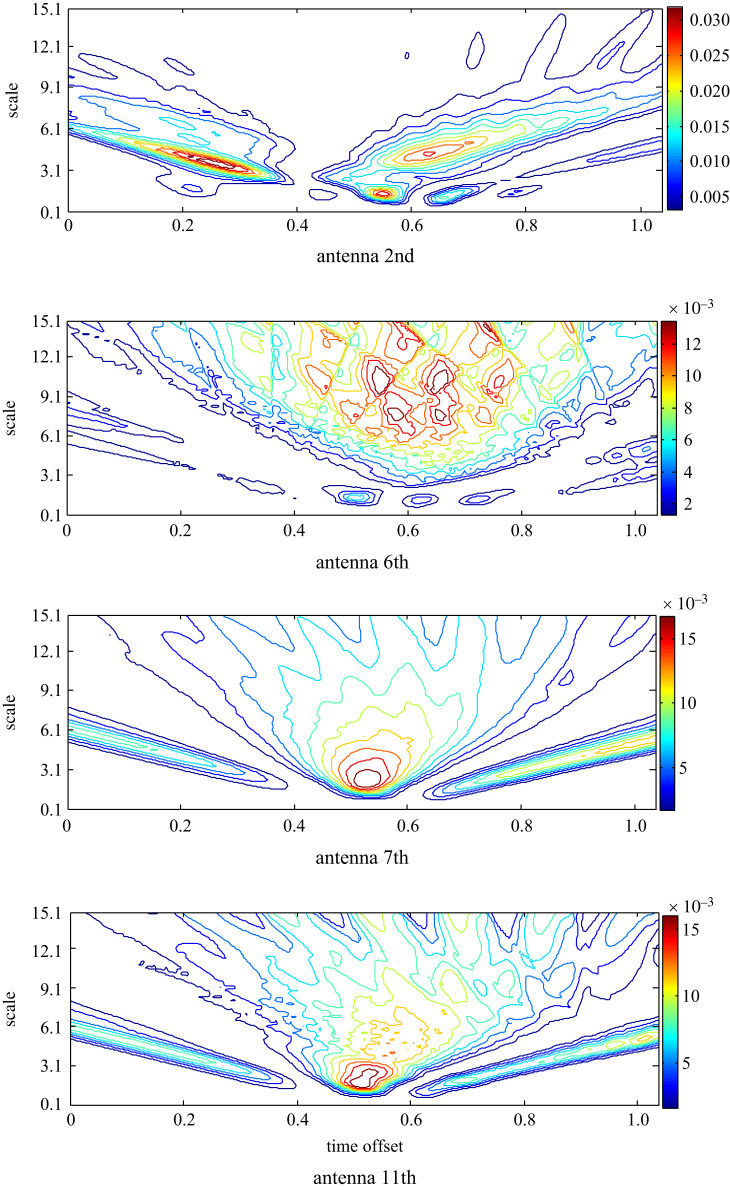
The scalogram of the matching filtered signals at the 2nd, 6th, 7th and 11th antennas after the pre-processing based on APP-5 to estimate the target response.

To evaluate the proposed approach App-5 that merges App-3 and App-4 to remove the background reflection, equation (3.6) is applied, and the scalogram to portray the distribution of the RWE in the scale–time plane is presented in figure 12. Similar to the results of App-4, the signal at the 2nd antenna records the highest RWE. While the 6th antenna records the lowest RWE. Further, the antennas that are far from the target showed a scalogram similar to that determined at the 2nd antenna with more than one hot spot, and the oppositely located 7th and 11th antennas portray similar scalograms. The 6th antenna has the most different scalogram where the wavelet energy is shown at higher scales and time offset compared with the other scalograms.

**Figure 13.**
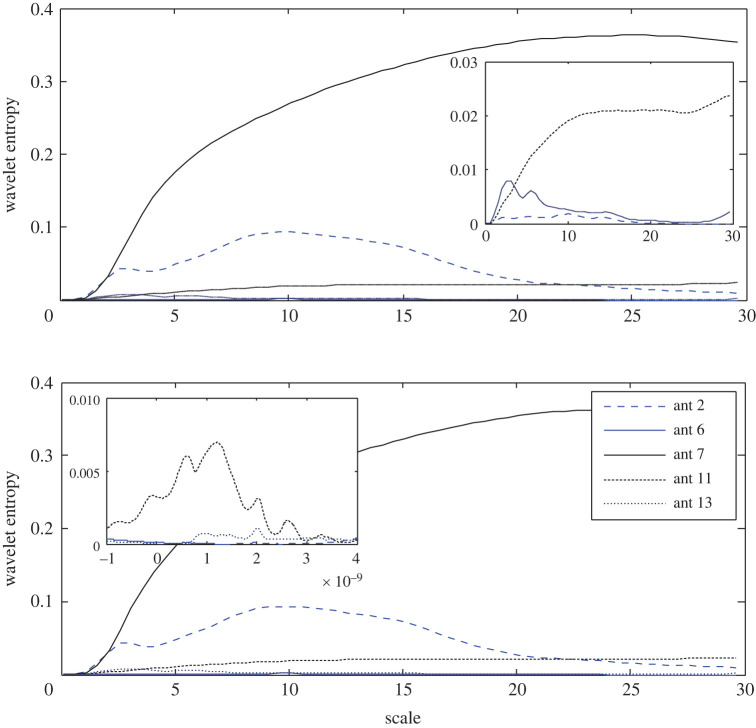
Wavelet Shannon entropy of the target response in the scale and time domain at the 2nd, 6th, 7th, 11th and 13th antennas using APP-5.

As represented in figure 13, the 7th antenna records the highest entropy and the 6th antenna records the lowest entropy in both domains in contrast to the earlier observed inverse proportion between the wavelet energy and entropy. For a clear view, the results with low entropy are zoomed in. Thus, the 6th antenna is expected to be the closer to a stroke.

The overall results of RWE and Shannon entropy using the five approaches are summarized in table 1, to show the inverse proportion between the wavelet energy and wavelet entropy, if it exists, based on the applied approach to remove the background reflection and estimate the response of interest. Using App-1 and App-3, the inverse proportion in two phases could be noticed, while using App-2 and App-4, those only in a single phase could be noticed. A direct relation is noticed using App-5 in a single phase.

**Table 1.** The outputs values of the wavelet matched filter using different approaches for background removal.

	lowest RWE	highest RWE	highest entropy	lowest entropy
App-1	2nd	7th	2nd	7th
App-2	2nd	6th	11th	6th
App-3	2nd	6th	2nd	6th
App-4	7th	2nd	7th	6th
App-5	6th	2nd	7th	6th

### 5.6. Distance between wavelet energy distributions

A novel tool called ‘distance between energy distributions’ in the wavelet domain is introduced in this work for detection purposes. It discriminates the wavelet energy at two opposite antennas either in the scale or time domain as given by

5.1 $$\omega_{\alpha i}=\underset{\tau }{\sum }\log_{2}\frac{{\left|C_{\alpha ,\tau }\right|}_{i}^{2}}{{\left|C_{\alpha ,\tau }\right|}_{\left(L+2-i)\mathrm{mod}(L+1\right)}^{2}}$$

and

5.2 $$\omega_{\tau i}=\underset{\alpha }{\sum }\log_{2}\frac{{\left|C_{\alpha ,\tau }\right|}_{i}^{2}}{{\left|C_{\alpha ,\tau }\right|}_{\left(L+2-i)\mathrm{mod}(L+1\right)}^{2}}$$

**Figure 14.**
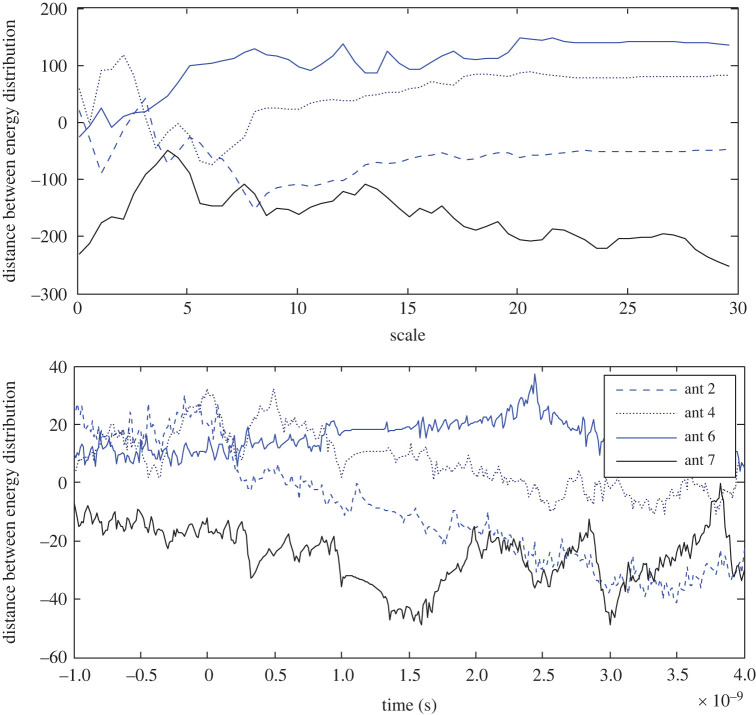
Distance between opposite energy distributions in the target response based on App-1 at the 2nd, 4th, 6th and 7th antennas in the scale and time domains.

**Figure 15.**
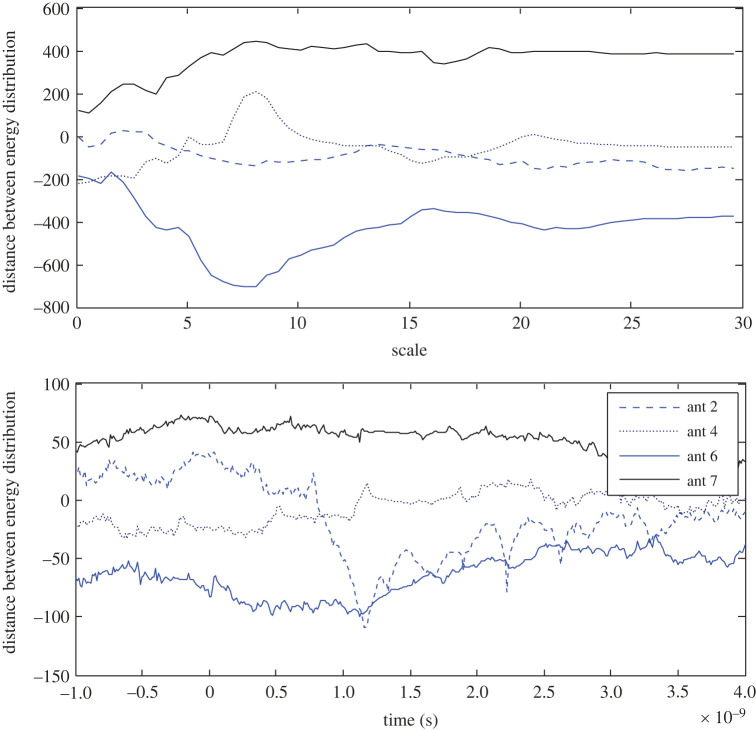
Distance between opposite energy distributions in the target response based on App-5 at the 2nd, 4th, 6th and 7th antennas in the scale and time domains.

Here $\omega_{\alpha i}=-\omega_{\alpha (L+2-i)\mathrm{mod}(L+1)}$ and the same is true for $\omega_{\tau }$. The tool is applied to test the distance between the opposite energy distribution in the target response with perfect App-1, and the new App-5, and the results are depicted in figures 14 and 15, respectively. For example, to find $\omega_{\alpha }$ at the 2nd antenna, the wavelet coefficients in the scale–time plane of the target response at the 2nd antenna and the opposite 16th antenna are applied in equation (5.1).

The procedure is applied at different antenna positions, as presented in the figures, and the results state that the closer the antenna is to the target, higher distances it records in both the scale and time domains. Using App-1, the results states that the wavelet energy at the 6th antenna is higher than that at the opposite antenna, except over the very low scales therefore a positive distance is determined. The energy at the 7th antenna is lower than that at the opposite 11th antenna, therefore the distance at the 7th antenna has negative values. The 7th antenna records the longest distance as an indication of its closeness to the stroke. Using App-5, the longest distance is found at the 6th antenna. The results based on App-5 state that the energy at the 7th/6th antenna is higher/lower than that at the opposite antenna 11th/12th. Using either App-1 or App-5, far-located antennas record bipolar distance in both the scale and time domain.

Furthermore, $\omega_{\alpha }$ > $\omega_{\tau }$ in both the figures, and the target response based on App-5 portrays larger discrimination between the opposite antennas, compared with the estimated response using App-1.

## 6. Conclusion

A computer-aided monitoring system, based on pattern recognition in the backscattered microwave signals, is studied as a cost-effective and portable tool to detect and localize brain stroke. The scattered fields at different antenna positions are processed through new multi-scale wavelet-matched filters. The filters are built with a new mother basic function dependent on the radiated microwave signal over the spectrum 1.1–3 GHz. The background reflection is removed via different approaches with various potentials for estimating the stroke response and reinforcing the detection process. The efficacy and accuracy to localize the stroke of the new proposed approach to estimate the target response were confirmed.

The filtered microwave signal in the time–scale plane is used to calculate the relative wavelet energy and the wavelet Shannon entropy that is well localized in space, content scales and time offset. A novel tool, based on calculating the distance between the wavelet energies at opposite antennas with respect to the line dividing the brain into right and left halves, is applied. The observed variance in the results around the head is a suitable monitor for diagnosis. The results demonstrate the effectiveness of the wavelet analysis in brain injury diagnosis and attract attention to the development of a microwave tool for the detection and localization of brain strokes.

## Data Availability

The data we used in this work is experimentally measured by a microwave system and owned by Dr Ahmed. The details of the system were published in B. J. Mohammed, A. M. Abbosh, S. Mustafa and D. Ireland, 2014, ‘Microwave System for Head Imaging’, IEEE Transactions on Instrumentation and Measurement, vol. 63, no. 1, pp. 117–123 [4], and the antenna design was published first in A. T. Mobashsher, B. J. Mohammed, S. Mustafa, and A. Abbosh, ‘Ultra wideband antenna for portable brain stroke diagnostic system,’ in 2013 IEEE MTT-S International Microwave Workshop Series on RF and Wireless Technologies for Biomedical and Healthcare Applications (IMWS-BIO), 9–11 December 2013, pp. 1–3 [14]. These data in wavelet domain are provided as electronic supplementary material, file. To access the original data that are recorded by the microwave system in the frequency domain, contact Dr Ahmed Mobashsher on Email: a.mobashsher@uq.edu.au. The signal processing for the proposed monitoring system in the current work is presented in the uploaded code as the second electronic supplementary material. The data are provided in electronic supplementary material [21].
